# Hydrated nucleus pulposus extrusion in dogs: correlation of magnetic resonance imaging and microsurgical findings

**DOI:** 10.1186/s13028-015-0151-x

**Published:** 2015-09-25

**Authors:** Mario Dolera, Luca Malfassi, Silvia Marcarini, Giovanni Mazza, Massimo Sala, Nancy Carrara, Roberto Vailati Facchini, Sara Finesso

**Affiliations:** La Cittadina Fondazione Studi e Ricerche Veterinarie, 26014 Romanengo, CR Italy

**Keywords:** Discal cyst, Intraspinal cyst, Extradural, Dorsal longitudinal ligament, Hydrated nucleus pulposus extrusion

## Abstract

**Background:**

Magnetic resonance imaging (MRI) patterns of canine cervical hydrated nucleus pulposus extrusion (HNPE) have been described by a few reports, but the correlation between microsurgical and MRI features has never been investigated. The aim of this study was to compare the MRI features of HNPE with microsurgical findings and cytological outcomes and also to investigate the anatomical and pathophysiological aspects of the disease.

**Methods:**

A prospective clinical study was conducted in 36 dogs suffering from HNPE. The diagnosis was based on high-field MRI findings of ventral extradural lesions, adjacent to the dorsal aspect of intervertebral discs, characterised by high signal intensity in T2-weighted sequences and hypointensity in T1-weighted sequences. MRI images were analysed with regard to the intervertebral space involved, the grading of spinal cord compression, the signal intensity and distribution of the material, and the thickness and signal intensity of the involved discs. All patients underwent microsurgical decompression and direct observations were recorded and films of the surgical procedure analysed.

**Results:**

The majority of patients had acute onset of clinical signs (78 %), the patient did not exhibit signs of pain in 75 % of dogs and neurological deficits varied from slight tetraparesis (33 %) to tetraplegia (28 %). The localization of the extruded disc material was ventral relative to the dorsal longitudinal ligament that was lifted dorsally and appeared intact at the site of compression. Direct microsurgical observations of the HNPE sites showed that extruded disc material was collected within the fibres of the dorsal longitudinal ligament. The consistency was gelatinous in 42 %, water-like in 33 %, and lumpy liquid in 25 % of cases. Cytological samples did not detect the presence of inflammation, bacteria, fungi, neoplastic cells or foreign material.

**Conclusions:**

Microsurgical features of HNPE suggest that the extruded disc is collected within the fibres of the dorsal longitudinal ligament and this may explain the typical MRI appearance of this disease. Further pathophysiological studies are needed to investigate why the cervical nucleus pulposus extrusion appears to occur without obvious trauma.

**Electronic supplementary material:**

The online version of this article (doi:10.1186/s13028-015-0151-x) contains supplementary material, which is available to authorized users.

## Background

The term intervertebral disc disease (IVDD) [1] can be used to summarise mechanisms by which a degenerating disc can cause pain and neurologic deficits, and it is defined as a localised displacement of the intervertebral disc beyond the normal anatomic limits of the disc [2]. Different types and mechanisms of disc degeneration can lead to different lesions, including the extrusion of the degenerate nucleus (“Hansen type I”) or the protrusion of the degenerating annulus into the vertebral canal (“Hansen type II”) [2]. The herniation of intervertebral disc material into the vertebral body is less commonly observed, and it has been named intravertebral disc herniation or Schmorl’s nodes [3]. Other types of disc herniation include the low volume-high velocity disc extrusion [4] and the so-called disc-associated intraspinal cysts or discal cysts, which have been described in both dogs and humans [1, 5–12]. Hydrated nucleus pulposus extrusion (HNPE) has been described recently in the cervical spine of 10 dogs that were presented for severe neurological signs [13]. The aim of this study was to investigate the relationship between microsurgical findings and high-field magnetic resonance imaging (MRI) of HNPE with particular regard to the anatomical localisation and pathophysiology of this disease.

## Methods

Between 2008 and 2012, a prospective clinical study on patients with suspected HNPE was conducted. The enrolment criteria were as follows: the presence of neurological signs referable to cervical spinal cord compression, the availability of the complete medical history and neurological examination, and an MRI-based diagnosis of suspected HNPE. Consent for a surgical treatment and cytological examination of the lesions was obtained.

Information regarding the onset, the presence of pain and the course of clinical signs were assessed by owner interview and analysis of data recorded in the medical record of each dog. The onset was classified as acute ≤24 h, subacute 24–48 h or chronic ≥48 h. Before the MRI examination thorough neurological examination was performed. Neurological signs were subjectively estimated by classifications into four categories (1) mild ambulatory tetraparesis (ability to walk without support), (2) severe ambulatory tetraparesis (inability to walk without support), (3) non-ambulatory tetraparesis complicated by mild dyspnoea (reduced function of the diaphragm and intercostal muscle that does not require respiratory assistance), and (4) tetraplegia complicated by severe dyspnoea (paralysis of the diaphragm and intercostal muscle that requires respiratory assistance).

The MRI examination was performed with a 1.5 T scanner (Intera 1.5 T, Philips Medical Systems, Eindhoven, The Netherlands) that was equipped with a phased array spinal coil. General anaesthesia was induced with intravenous propofol (Fresenius Kabi Italia Srl, Isola della Scala, Verona, Italy) followed by oro-tracheal intubation and anaesthesia was maintained with a mixture of isoflurane (IsoFlo, Abbott House, Berkshire, United Kingdom) in oxygen and medical air. For each animal, the following scans were performed: a fluid-only thick slab sagittal scan (TR 8000 ms, TE 900 ms, slice thickness 40 mm, matrix 512, NEX 1, FOV 450 mm, acquisition time 0.13 min); a Turbo Spin Echo (TSE) T2-weighted (T2-W) two dimensional (2D) sagittal scan (TR 3500 ms, TE 130 ms, slice thickness 2 mm, matrix 1024, NEX 2, FOV 530 mm, acquisition time 4.5 min); a Fast Field Echo (FFE) T1-weighted (T1-W) 2D transverse scan (TR 340 ms, TE 3.5 ms, slice thickness 3 mm, matrix 512, NEX 3, FOV 120 mm, acquisition time 3.5 min); and a TSE T2-W 2D transverse scan (TR 3500 ms, TE 130 ms, slice thickness 2 mm, matrix 512, NEX 2, FOV 200 mm, acquisition time 3.8 min) of the entire cervical spine. When the anaesthetic protocol permitted, additional sequences were performed, including a T2-W Fluid Attenuated Inversion Recovery (FLAIR) 2D sagittal scan (TR 6000 ms, TE 100 ms, TI 2000 ms, slice thickness 3 mm, matrix 256, NEX 2, FOV 250 mm, acquisition time 4.8 min), a T2-W Short Tau Inversion Recovery (STIR) 2D sagittal scan (TR 2500 ms, TE 50 ms, TI 150 ms, slice thickness 3 mm, matrix 512, NEX 2, FOV 250 mm, acquisition time 4.6 min), and a T1-W Spin Echo (SE) transverse 2D scan (TR 300 ms, TE 20 ms, slice thickness 2 mm, matrix 512, NEX 3, FOV 530 mm, acquisition time 3.6 min) before and after the intravenous administration of 0.15 mmol/kg gadodiamide (Omniscan, GE Healthcare, Milan, Italy), which was injected into the left cephalic vein.

The following MRI features were assessed: spinal cord compression and related grading, the intervertebral disc spaces that were involved, the signal of the compressive material, the localisation and distribution of the material relative to the dorsal longitudinal ligament, the width of the subarachnoid space at the lesion site, the spinal cord signal in T2-W sequences, and the thickness and MRI signal of the intervertebral disc adjacent to the compression. A previously described method was used to evaluate spinal cord compression [14]. Briefly, on cross-sectional T2-W images the area of the spinal cord at the point of maximum compression was measured by tracing the outline of the cord. The region of normal spinal cord closest cranially to the site of compression was identified and the cross-sectional area of the uncompressed spinal cord was measured. These measurements were used to calculate the grade of compression as percentages with respect to the normal spinal cord [14]. The width of the subarachnoid space was subjectively evaluated by the radiologist on sagittal scans comparing the dorsal and ventral subarachnoid space at the lesion site with that of uninvolved spinal cord tracts. The spinal cord signal was evaluated on sagittal T2-W images comparing the signal at the point of maximum compression with the signal of the spinal cord adjacent to other cervical intervertebral discs that were considered to have normal appearance. The thickness and MRI signal of the intervertebral disc were subjectively evaluated on sagittal images by the radiologist, who visually compared the disc corresponding to the compression with the signal of other cervical intervertebral discs that were considered normal.

Surgical treatment was performed immediately after MRI examination; a surgical microscope was used (NC-4, Carl Zeiss, D-73446 Oberkochen, Germany) for all procedures. Each patient was placed in dorsal recumbency and the neck was positioned in mild extension. After blunt dissection of the pre-vertebral muscles, a ventral slot was created at the affected intervertebral space, using the standard described technique and by the means of a high-speed burr. The slot extended no more than the caudal third of the cranial vertebra to the cranial third of the caudal vertebra, and its width did not exceed a third of the width of the vertebral bodies [15]. A microdiscectomy was performed and the fenestration of the intervertebral disc allowed the visual inspection of the dorsal portion of the annulus fibrosus in all the dogs. After cutting the annulus fibrosus with a Kaspar n. 857 blade (Braun, Tuttlingen, Germany) the dorsal longitudinal ligament was sliced, and the liquid content was removed and collected (see Additional file 1). The microsurgical findings included the macroscopic structure of the lesion before surgical excision; the characteristics of the compressing material (colour, transparency, texture, and output pressure); the localisation of the lesion and the specific relationship with the longitudinal ligament; the presence of epidural haematomas; and the macroscopic appearance of the spinal cord. The compressive material was collected by suction. The cytological samples were obtained by direct smear on a slide while collecting the evacuated material during surgery; these samples were stained with Diff-Quik staining solutions (Tektron, Bomheim, Germany).

In the postoperative period, each animal was evaluated regarding the timing and quality of functional ambulatory and respiratory recovery. Information on the clinical status of the patients during the follow-up period was obtained by periodic clinical examinations and/or telephone interviews with the animals’ owners.

## Results

Thirty-six dogs (21 males, 15 females; 18 small-sized mixed breeds, 4 Pinschers, 4 West Highland White Terriers, 3 Poodles, 3 Yorkshire Terriers, 2 large-sized mixed breeds, 1 Maltese, 1 Pekingese) fulfilled the inclusion criteria. The mean body weight was 10.6 kg, with standard deviation of ±6, range from 2.5 to 34.2 kg.

### Anamnesis

The onset of clinical signs was hyperacute (within a few seconds) in 5/36 cases (14 %), acute (minutes to hours) in 28/36 cases (78 %), and gradual (days) in 3/36 cases (8 %). Patients did not exhibit pain at the onset in 32/36 of cases (89 %) while 4/36 dogs showed presence of pain (11 %).

### Clinical presentation

At the time of the clinical examination 27/36 (75 %) patients did not appear painful and 9/36 (25 %) were mildly painful. The course of pain was stable in 32/36 cases (89 %) and progressive in 4/36 cases (11 %). Only 1/36 animals exhibited pain without neurological deficits (3 %). Neurological classification resulted in: 12/36 dogs showing mild ambulatory tetraparesis (33 %), 13/36 dogs showing severe ambulatory tetraparesis (36 %), 5/36 dysplaing non-ambulatory tetraparesis complicated by mild dyspnoea (14 %), 5/36 showing tetraplegia (14 %) that was complicated by severe dyspnoea in 2/36 dogs (6 %).

### MRI findings

**Fig. 1:**
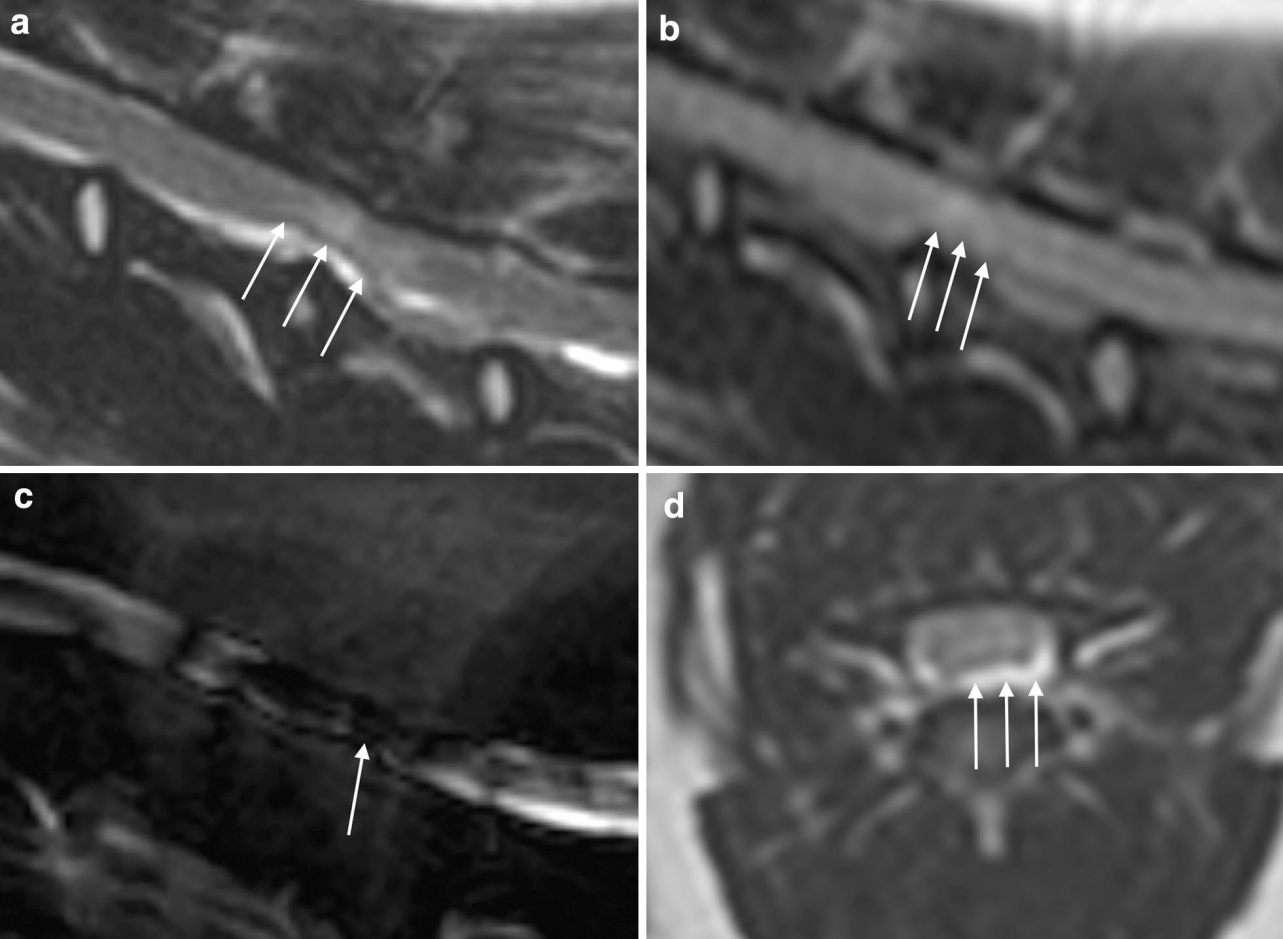
Magnetic resonance imaging images from a dog presented with ambulatory tetraparesis. HNPE at C4-C5 determining mild neural compression. **a** Sagittal T2-W turbo spin echo (TSE) sequence where the hydrated material shows T2 signal similar to the CSF; the *arrows* indicate the point at which the longitudinal ligament rises from the floor of the spinal canal. **b** Sagittal T2-W fluid attenuated inversion recovery (FLAIR) sequence; the *arrows* indicate the hyperintense, poorly defined region at the spinal cord segment corresponding to the HNPE. **c** Fluid-only thick slab sagittal scan; the *arrows* indicate the area of reduced CSF signal corresponding to the spinal cord compression. **d** Transverse T2-W TSE sequence; the *arrows* indicate the compressive material

**Fig. 2:**
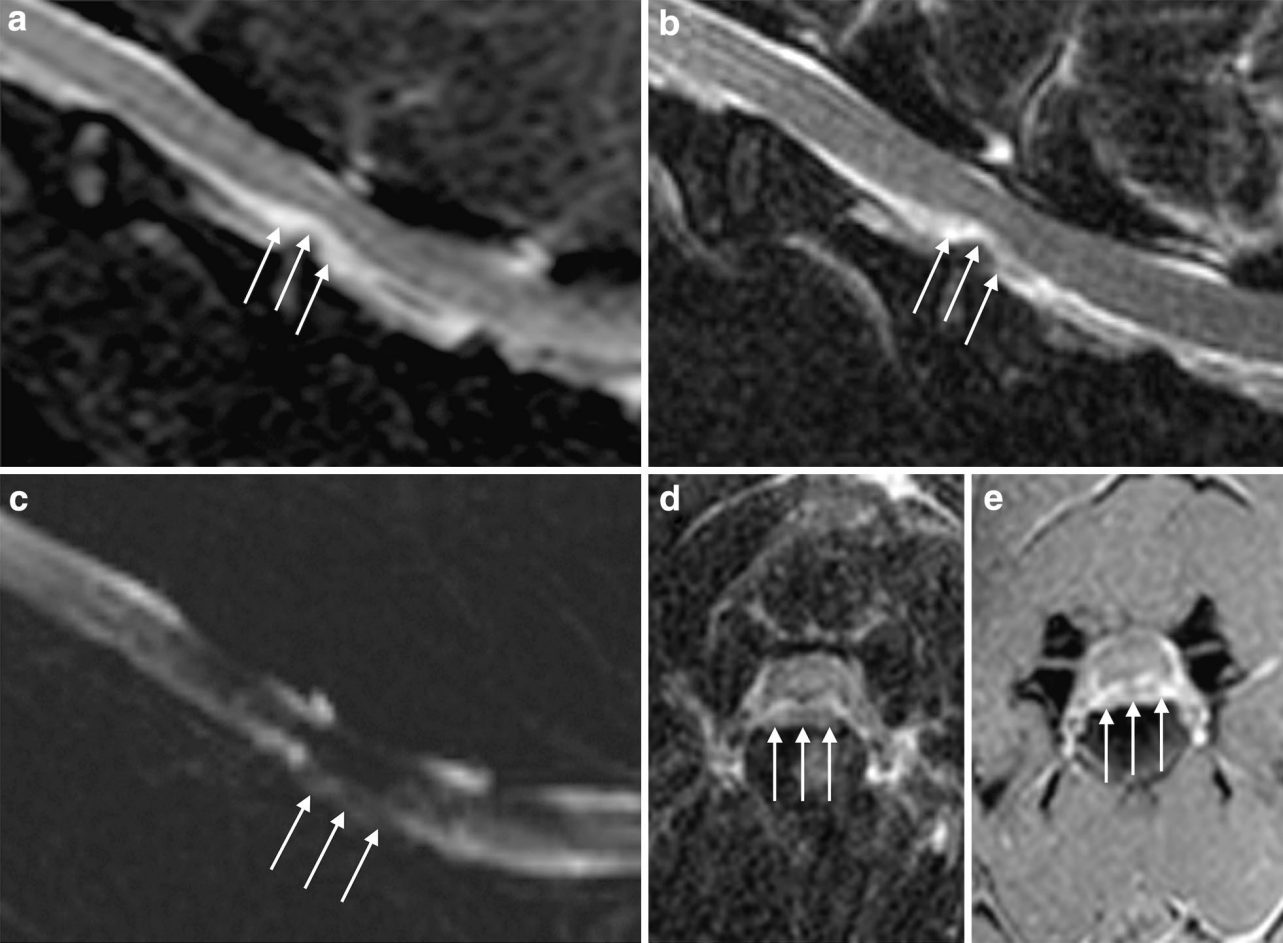
Magnetic resonance imaging images from a dog presented with non-ambulatory tetraparesis. HNPE at C4-C5 determining moderate-severe neural compression. **a** Sagittal T2-W short tau inversion recovery (STIR) sequence; with fat-suppression the lesion maintains the characteristic T2 high signal. **b** Sagittal T2-W turbo spin echo (TSE) sequence; the *arrows* indicate the compressive material that is inhomogeneously isointense compared with CSF. **c** Fluid-only thick slab sagittal scan; please note the disomogeneous area of hypointensity at the site of compression (CSF). **d** Transverse TSE T2-W scan; the *arrows* show the HNPE with quite symmetrical distribution. **e** Transverse T1-W FFE sequence after contrast medium administration; please note the peripheral enhancement of the compressive lesion

HNPE was identified as ventral extradural lesions in the vertebral canal adjacent to the intervertebral disc (Figs. 1, 2). All the lesions had high signal intensity in T2-W sequences; in particular, they were isointense to cerebrospinal fluid (CSF) in 33/36 cases (92 %) and isointense with hypointense foci in 3/36 (8 %). When T2-W STIR sequence was performed (15/36) the compressive material displayed high signal intensity, that was isointense to CSF in all patients (Fig. 2a). On T2-W FLAIR sequence (acquired in 27/36 dogs) HNPE could not be identified and a hyperintense, poorly defined region was observed at the spinal cord segment corresponding to the HNPE (Fig. 1b). The signal intensity of HNPE was low in T1-W sequences, appearing isointense to CSF in 28/36 (78 %) cases or slightly hyperintense in 8/36 (22 %) cases. When the anaesthetic session could be extended, T1-W images after contrast administration were acquired (in 7/36 patients) and showed marginal enhancement around the lesion (Fig. 2e). In the majority of the patients (28/36) the compressive material was not clearly identifiable in fluid-only thick slab sagittal scan but interruption of the CSF signal was evident at the site of compression (Fig. 1c). In 6/36 dogs the same sequence pointed out a diffuse hypointensity of the spinal cord at the involved site (Figs. 1c, 2c). The hydrated material of suspected disc origin could be distinguished by its signal intensity, which was similar to the CSF signal in both T1-W and T2-W images (Figs. 1, 2). On MRI, the compressing material appeared to be localised ventrally with respect to the dorsal longitudinal ligament with distribution ranging from symmetrical to slightly lateralized on transverse planes. The ligament was evaluated on T2-W images; in midsagittal sections, the structure was noticeable as a continuous linear region that was hypointense relative to CSF and extruded disc material, and lifted up at the site of compression (Fig. 1a). The width of the subarachnoid space was reduced at the level of involvement and it was lost in 7/36 patients. The involved vertebral spaces were C4–C5 in 32/36 cases (89 %) and C3–C4 in 4/36 (11 %). The compression ranged from 13.5 to 57.2 % with a median compression of 36.5 %. The spinal cord signal at the point of maximum compression was normal in 31/36 patients (86 %) and slightly T2 hyperintense in 5/36 cases (14 %). The thickness of the discs corresponding to the compression was slightly reduced in 27/36 cases (75 %) and normal in 9/36 cases (25 %); additionally, the MRI signal of the discs corresponding to the compression was isointense to other cervical intervertebral discs in 30/36 cases (83 %) and slightly reduced in T2-W sequences in 6/36 cases (17 %).

### Surgical findings

**Fig. 3:**
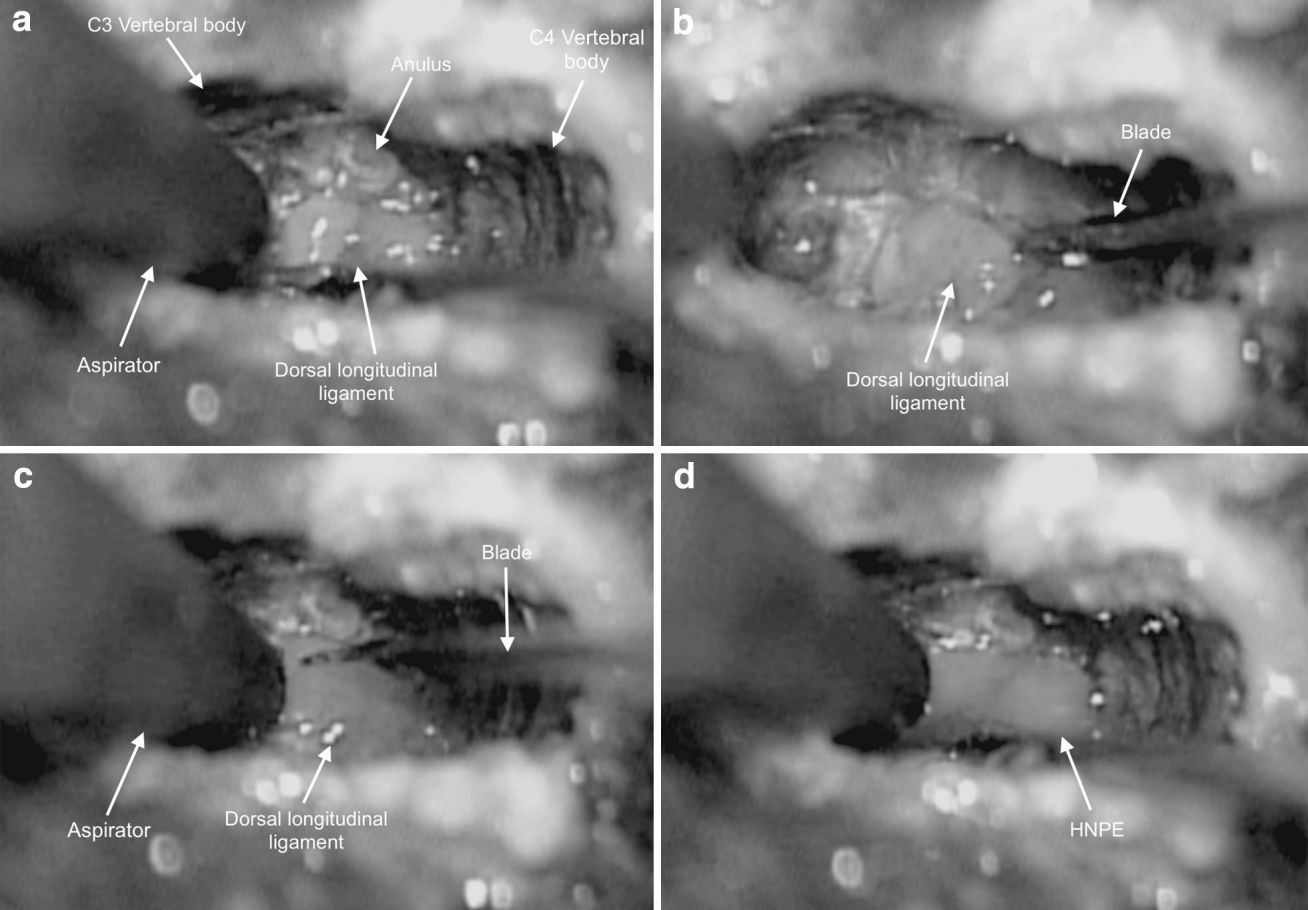
Four steps of the microsurgical decompression. For each image cranial is to the *left*, caudal is to the *right*, the *right side* is below, the *left side* is above. **a** The dorsal longitudinal ligament is seen through the ventral slot. **b** The incision of the dorsal longitudinal ligament using a microsurgical blade. **c** The resection of dorsal longitudinal ligament can be seen. **d** The ligament has been sheared off and the HNPE leaked out with strong spurt, suggesting the material was under pressure

The most meaningful sequences of surgery are shown in Additional file 1. The systematic use of a surgical microscope enabled direct observations of the location and consistency of the lesions. First, in all cases a standard intervertebral ventral slot with microdiscectomy was performed. In the dorsal part of the annulus, a single fissure was detected by visual inspection. Once the dorsal part of the annulus was removed, the dorsal longitudinal ligament was visualised (Fig. 3). In all the patients slicing the ligament allowed the compressing material to leak out with a strong spurt, suggesting the material was under pressure. In 17/36 patients thin septa within the ligament were observed at the lesion sites. In 23/36 patients a small incision was needed to allow the material to leak out, whereas in the remaining 13/36 cases the ligament needed to be sheared off entirely to permit removal (Fig. 3). A clean dural plane was present ventrally between the lesion and the spinal cord, and all lesions were considered extradural. The colour of the compressive material was light grey in 21/36 cases (58 %), colourless in 14/36 (39 %), and white in 1/36 (3 %) (Fig. 3). The material was turbid in 25/36 cases (69 %), transparent in 10/36 (28 %) and opaque in 1/36 (3 %). The texture was a gelatinous liquid in 15/36 animals (42 %), water-like in 12/36 (33 %), and lumpy liquid in 9/36 (25 %). The spinal canal was probed and optically examined, and neither epidural haematomas nor spinal cord haemorrhage were observed.

The cytological specimens were characterised by poor cellularity and the presence of abundant basophilic amorphous material ranging from finely granular to coarse. Within the sample, neutrophils, spindle cells of mesenchymal origin without atypical characteristics and some haemosiderin-laden histiocytes were rarely observed. Crystalline nonstaining structures mixed with the amorphous material were also found rarely. Bacteria, fungal pathogens or atypical cells were not detected in any samples.

### Follow-up

Patients regained ambulatory status within 1 week in 26 cases (72 %), within 4 weeks in nine cases (25 %), and after 4 weeks in one case (3 %). In dogs with respiratory impairment, normal respiratory function was regained following anaesthesia. The prospective follow-up period was limited to 1 year. During that time eight dogs underwent physical examination and were found to be ambulatory. In 28 cases the owners were contacted via telephone and the patients were found to be alive and ambulatory. No dogs developed other disc extrusions within 1 year.

## Discussion

Several cystic lesions of the spine have been described in dogs [16–22]. Arachnoid diverticulum cysts, meningeal cysts, synovial cysts and dermoid-epidermoid cysts [16–22] have been described on imaging as having different features than HNPE. The first report of a lumbar lesion with a cystic-like appearance and connection to a disc was described in 2007 in a purebred Rottweiler dog [6]. The compressive material was detected by high-field MRI and confirmed at surgery. It was defined as a “degenerative intraspinal cyst associated with an intervertebral disc” or an “atypical spinal synovial cyst”, suggesting a similarity with the most frequently described classic synovial cysts, which have been reported both in humans and in dogs [6]. In 2008, a study that described the low field MRI and surgical findings of intraspinal disc-associated cysts in seven dogs was published [5]. Although the findings were similar to the description provided in 2007, the adjective “degenerative” was replaced with “ventral” in the definition of the lesion to note the topography of the lesion rather than its uncertain pathogenesis [6]. In 2010 another study [7] described the “spontaneous regression of a cervical intraspinal cyst” in a Border Collie. The authors hypothesized that the intraspinal cysts originated from a disc-associated nuclear prolapse, followed by bleeding from the epidural venous plexus. However, due to the spontaneous resolution of the described case, the authors were unable to substantiate their hypothesis with surgical or pathological findings. Beltran and others’ retrospective publication on ten dogs is the most extensive work devoted to this topic [13].

The physiopathology of HNPE is still not completely understood. The acute, compressive extrusion of hydrated disc material has not been experimentally induced and accurate experimental model may not be possible due to the complex anatomy of the small structures involved.

Moreover, the generally high rate of recovery from the disease, even with a conservative-medical therapy, precludes post-mortem examinations [7, 10, 13]. The liquid texture of the compressive material does not allow histological examination, and its poor cellularity does not fit the aim of histopathology. The previously reported MRI features together with the results from the present study describe the criteria for a consistent imaging-based diagnosis. On MRI, spinal extradural compressive lesions that are ventrally located, adjacent to the corresponding intervertebral disc, with fluid signal and symmetrical to the spinal cord on the midline ventral, are considered to be HNPE.

Because the pathological mechanisms of HNPE have not yet been elucidated, the aim of this prospective study was to readdress MRI and clinical features by microsurgical confirmatory diagnosis and direct anatomical observations. A previous report indicates that the relationship between an extruded nucleus pulposus or protruded annulus and the spinal cord can be adequately examined with cross-sectional MRI images [1]. The authors of the paper state that different types of disc-associated lesions can be defined, including bulging, protruding, and extruding [1]. Moving forward from these previous verifications, in our research HNPE characteristics and localization have been defined through the following evidence. First, the presence of a lesion that was isointense to CSF on T1-W images and consistently hyperintense on T2-W images was supportive of the compressive material having a liquid consistency. Second, the absence of CSF flow from the slot at the time of surgical decompression excludes a possible connection between meningeal layers and fluid collection in the epidural space. In addition, no infectious agents were detected upon cytological examination.

We can definitively agree on MRI examination reliability in identifying the correct anatomical localisation but only with respect to the spinal canal. In fact lesions were extradural but the MRI appearance suggested they were located ventral to the dorsal longitudinal ligament. With this anatomical localisation and a ventral surgical approach, the compressive lesions should be accessible without cutting the dorsal longitudinal ligament. In contrast at surgery the ligament had to be dissected to permit removal in all the patients (Additional file 1). This finding is of interest because it adds the understanding of HNPE peculiar localization and it comes in agreement with the previously demonstrated low sensitivity of MRI in differentiating between subligamentous versus extraligamentous disc material [23]. The false-negative MRI evaluation, on which HNPE seemed not to breach the ligament, could also be ascribed to chemical-shift artefacts, with special regard to herniations of small size [23]. In addition to the need of cutting the ligament, the leaking of the compressive material that appeared to be under pressure was observed in all the cases of our cohort and the same finding has been described in other previous studies [5].

The authors believe that the dorsal longitudinal ligament allows extruded disc material to distribute within its thickness, providing an explanation for the microsurgical findings and the MRI subligamentous appearance. The characteristic ventral and symmetrical localisation of HNPE, as reported in literature, could also be explained by this anatomical relationship.

The thickness and the signal intensity of the involved discs are discussed. On MRI, the majority of the discs corresponding to HNPE showed slight reduction of the thickness (75 %), whereas the T2 signal intensity was isointense to other cervical intervertebral discs in 83 % of the cases and slightly reduced in the remaining 17 % of the cases. Therefore, the nucleus pulposus of such discs was not degenerate, and the extrusion may only involve a little, not or only mildly degenerate part of the nucleus pulposus. The reason for the intraligamentous localisation could partially arise from the consistency of the extruded material: rather than tearing the fibres of the longitudinal ligament, the liquid herniation parted them giving rise to the septa observed at surgery. In this study discography was avoided because of the potential risk for neurological complications, but the fissure in the annulus fibrosus observed during surgery could demonstrate the communication between the disc and the vertebral canal.

MRI post-contrast sequences were not considered mandatory because no clear benefits have been demonstrated from administering contrast medium to dogs with disc herniation [23]. The finding of marginal post-contrast enhancement in patients that underwent contrast medium administration suggests a cyst-like structure, but a well-defined cystic wall was not detected in multiplanar images. Furthermore, surgical observations did not support a cyst-like lesion. The authors suggest that the observed peripheral enhancement could be attributed to contrast extravasation from the meningeal vessels, as previously documented in humans [24]. In this case the enhancement could arise from fenestrated neovascularisation due to the inflammatory and compressive nature of the condition [24, 25]. Another hypothesis, based on MRI studies in humans, is that spinal compression may have produced internal vertebral venous plexus congestion [26]. Images from the seven patients with peripheral enhancement are supportive of venous plexus anatomical localisation. Vertebral angiography could further clarify this point in the future.

Fluid-only thick slab sagittal scan is usually performed by the authors of this paper as the starting sequence for spinal protocol since it requires very short time and it allows a quick identification of an acute spinal cord compression. The HNPE cannot be clearly identified in this sequence, but reduced or interrupted CSF signal was observed, probably due to the reduction of dorsal subarachnoid space caused by the spinal cord displacement. In some patients the same sequence disclosed heterogeneous hypointensity of the spinal cord and surrounding soft-tissues at the site of HNPE that can be ascribed to the inflammation and the related increased cellularity.

The presence of not well defined hyperintense signal of the spinal cord on FLAIR sequence should be considered as a presumptive and supportive finding of spinal cord oedema at the site of HNPE, however, the inability of this sequence in identifying the compressive material leads to the consideration that no additional information are provided by FLAIR images.

The clinical findings of our cases deserve further discussion. Previously described [5] discal cysts have resulted in clinical signs that are indistinguishable from acute disc prolapse; however, the onset, timing and pain characteristics were not examined in detail. The clinical signs of our group were similar to those of dogs with acute non-compressive nucleus pulposus extrusion [27] and are consistent with previously described HNPE [13]. In particular, tetraparesis of varying degrees without cervical discomfort at presentation, the abrupt onset and the lack of progression appear to be the main clinical features shared with ischaemic myelopathy [28], high velocity low-volume disc disease [29], traumatic intervertebral disc extrusion [30] and HNPE. In the authors’ opinion the rapid onset of clinical signs excludes the hypothesis that the observed lesions were pre-existing cysts that gradually formed, according to the mechanism proposed in 1999 for intraspinal extradural cysts [8].

In contrast, high velocity low-volume disc disease and traumatic disc extrusions differ from HNPE because of the concurrence of traumatic events or intense exercise at the onset and the slight to moderate cervical cord compression. The absence of lateralisation in neurological signs can be considered a useful criterion of differentiation from ischaemic myelopathy [28] and traumatic intervertebral disc extrusion [31]. Several hypotheses may be proposed to explain the low incidence of pain at clinical examination. Because the prolapsed material is very soft, the compressive effect exerted on neural structures may be somehow reduced. In addition, the integrity of the longitudinal ligament, precluding direct contact with the meninges and root ganglions, could mitigate the painful effect.

The majority of the observed patients were small in size, and the most frequent location was C4–C5 followed by C3–C4. The same sites, with an additional C5–C6 localisation, have been reported in acute non-compressive nucleus pulposus extrusion [26]; however, the reasons for this localisation are unknown.

Taking into account the severity of neurological presentation and the percentage of spinal cord compression, which was over 25 % in more than 91 % of the patients (33/36), surgical decompression was performed in all patients (36/36). The results showed regained ambulatory status within four weeks in 97 % of the patients. The previous study on HNPE suggested that the surgical treatment depended on the degree of spinal cord compression and the severity of clinical presentation [13]. In cases without signs of neurological impairment, observation may be recommended [7, 32]. However, surgery is the standard treatment for high-grade compression or when myelopathy is detected [33, 34].

In this study cytological examination of the hydrated materials was systematically performed. Although the variability in cytological findings from extruded canine disc material may not make it a reliable tool to differentiate disc herniation from other pathological conditions [35], the authors believe that the absence of bacteria, fungal pathogens and atypical cells should be taken into consideration. The lack of histological evaluation is shared with Beltran and others’ works but, as previously noted for intraspinal cysts [5], the leakage of material that occurred during surgery prevented histopathological staging.

There are many points of agreement between the present work and the results of the mentioned Beltran’s group retrospective study. In particular, we found many similarities with their clinical presentations because the majority of their dogs did not have cervical discomfort, although all of them were non-ambulatory. The absence of trauma or intense exercise in the medical history and the presence of respiratory complications in some patients are also in agreement. The dogs included in Beltran’s work were both non-chondrodystrophic and chondrodystrophic breeds, but, as a differentiating fact, the median bodyweight ranged from 10 to 37 kg, whereas the majority of our group (34/36) was composed of small-size dogs. The MRI findings from their ten patients showed both similarities and differences compared with our outcomes. The localisation of the compression, signal intensity characteristics and contrast enhancement were similar, while the focal intramedullary high signal intensity on T2-W images was not present in any of the 36 dogs in this study.

## Conclusions

Microsurgical direct observations together with MRI features, cytological examination and clinical aspects contributed substantially to the definition of HNPE. This study provides evidence that the hydrated part of the nucleus pulposus can extrude within the fibres of the dorsal longitudinal ligament and cause spinal cord compression. Further studies are needed to clarify the pathophysiology of this cervical spine disease.

## Additional file

10.1186/s13028-015-0151-x Film clip of the most meaningful sequences of a surgical treatment. The clip was obtained by the camera integrated into the surgical microscope (NC-4, Carl Zeiss, D-73446 Oberkochen, Germany). The ventral-slot technique has been performed with the patient positioned in perfect dorsal recumbency with the neck in mild hyperextension. A ventral midline incision was made and the the longus colli, sternohyoideus, and sternocephalicus muscles were dissected. Recording starts when the annulus fibrosus is approached in the contest of the exposed longus colli muscles. The fenenstration of the annulus fibrosus can be appreciated and the following removal of the incised, ventral part of the annulus fibrosus with a mosquito forceps is cleary shown. By the mean of a high-speed burr a ventral mini-slot is performed; note that the slot extended from less of the caudal third of C4 to less of the cranial third of C5. The annulus is therefore completely removed followed by additional cutting of the dorsal residual part. It is noticeable the full opening of the spinal canal and the exposure of the dorsal longitudinal ligament. The first cutting of the annulus fibrosus with a Kaspar n. 857 blade (Braun, Tuttlingen, Germany) is recorded and it is followed by further cutting and leaking out of the colourless, transparent, liquid compressive material. In the final sequence a surgical hook is used to slice the intraligamentous septa and to probe the spinal canal.

## Abbreviations

CSF: cerebrospinal fluid
FFE: FAST field echo
FLAIR: fluid attenuated inversion recovery
HNPE: hydrated nucleus pulposus extrusion
IVDD: intervertebral disc disease
MRI: magnetic resonance imaging
SE: spin echo
TSE: turbo spin echo
T1-W: T1-weighted
T2-W: T2-weighted

## References

[1] Jeffery ND, Levine JM, Olby NJ, Stein VM (2013). Intervertebral disk degeneration in dogs: consequences, diagnosis, treatment and future directions. J Vet Intern Med.

[2] Fardon DF, Milette PC (2001). Combined Task Forces of the North American Spine Society, American Society of Spine Radiology, and American Society of Neuroradiology. Nomenclature and classification of lumbar disc pathology. Recommendations of the Combined Task Forces of the North American Spine Society, American Society of Spine Radiology, and American Society of Neuroradiology. Spine J.

[3] Gaschen L, Lang J, Haeni H (1995). Intravertebral disk herniation (Schmorl’s Node) in five dogs. Vet Radiol Ultra.

[4] Laitinen O, Puerto D (2005). Surgical decompression in dogs with thoracolumbar intervertebral disc disease and loss of deep pain perception: a retrospective study of 46 cases. Acta Vet Scand.

[5] Konar M, Lang J, Fluhmann G, Forterre F (2008). Ventral intraspinal cysts associated with intervertebral disc: magnetic resonance imaging observations in seven dogs. Vet Surg.

[6] Penning V, Benigni L, Steeves E, Capello R (2007). Imaging diagnosis-degenerative intraspinal cyst associated with an intervertebral disc. Vet Radiol Ultrasound.

[7] Kamishina H, Ogawa H, Katayama M, Yasuda J, Sato R, Tohyama K (2010). Spontaneous regression of a cervical intraspinal cyst in a dog. J Vet Med Sci.

[8] Kono K, Nakamura H, Inoue Y (1999). Intraspinal extradural cysts communicating with adjacent herniated disks: imaging characteristics and possible pathogenesis. Am J Neuroradiol.

[9] Chiba K, Toyama Y, Matsumoto M (2001). Intraspinal cyst communicating with the intervertebral disc in the lumbar spine: discal cyst. Spine J.

[10] Demaerel P, Eerens I, Goffin J (2001). Spontaneous regression of an intraspinal disc cyst. Eur Radiol.

[11] Jeong GK, Bendo J (2003). Lumbar intervertebral disc cyst as a cause of radiculopathy. Spine J.

[12] Kishen T, Shetty A, Rajasekaran S (2006). Variant of a lumbar disc cyst in a 13 year-old girl: a case report. J Orthop Surg (Hong Kong).

[13] Beltran E, Dennis R, Doyle V, De Stefani A, Holloway A, de Risio L (2012). Clinical and magnetic resonance imaging features of canine compressive cervical myelopathy with suspected hydrated nucleus pulposus extrusion. J Small Anim Pract.

[14] Ryan TM, Platt SR, Llabres-Diaz FJ, McConnell JF, Adams VJ (2008). Detection of spinal cord compression in dogs with cervical intervertebral disc disease by magnetic resonance imaging. Vet Rec.

[15] Coates JR, Hoffman AG, Dewey CW. Surgical approaches to the spine. In: Slatter D, editor. Textbook of small animal surgery, 3 edn. Saunders: Philadelphia; 2003. pp. 1148–63.

[16] Tomlinson J, Higgins R, LeCouteur R (1988). Intraspinal epidermoid cyst in a dog. J Am Vet Med Assoc.

[17] Hardie R, Linn K, Rendano V (1996). Spinal meningeal cyst in a dog: a case report and literature review. J Am Anim Hosp Assoc.

[18] Lambrechts N (1996). Dermoid sinus in a crossbred Rhodesian ridgeback dog involving the second cervical vertebra. J S Afr Vet Assoc.

[19] Levitski R, Chauvet A, Lpsitz D (1999). Cervical myelopathy associated with extradural synovial cysts in 4 dogs. J Vet Intern Med.

[20] Shamir M, Lichovsky D, Aizenberg I (1999). Partial surgical removal of an intramedullary epidermoid cyst from the spinal cord of a dog. J Small Anim Pract.

[21] Perez B, Rollan E, Ramiro F (2000). Intraspinal synovial cyst in a dog. J Am Anim Hosp Assoc.

[22] Rylander H, Lipsitz D, Berry W (2002). Retrospective analysis of spinal arachnoid cysts in 14 dogs. J Vet Intern Med.

[23] Uhlenbrock D. Degenerative disorders of the spine. In: Uhlenbrock D, editor. MR Imaging of the Spine and Spinal Cord. Stuttgart: Thieme; 2004. pp. 198–200.

[24] Braun P, Kazmi K, Nogues-Melendez P, Mas-Estellés F, Aparici-Robles F (2007). MRI findings in spinal subdural and epidural hematomas. Eur J Radiol.

[25] Suran JN, Durham A, Mai W, Seiler GS (2010). Contrast enhancement of extradural compressive material on magnetic resonance imaging. Vet Radiol Ultrasound.

[26] Morikawa M, Sato S, Numaguchi Y, Mihara F, Rothman MI (1996). Spinal epidural venous plexus: its MR enhancement patterns and their clinical significance. Radiat Med.

[27] De Risio L, Adams V, Dennis R, McConnell FJ (2009). Association of clinical and magnetic resonance imaging findings with outcome in dogs with presumptive acute noncompressive nucleus pulposus extrusion: 42 cases (2000–2007). J Am Vet Med Assoc.

[28] De Risio L, Platt SR (2010). Fibrocartilaginous embolic myelopathy in small animals. Vet Clin North Am Small Anim Pract.

[29] Lu D, Lamb CR, Wesselingh K, Targett MP (2002). Acute intervertebral disc extrusion in a cat: clinical and MRI findings. J Feline Med Surg.

[30] Chang Y, Dennis R, Platt SR, Penderis J (2007). Magnetic resonance imaging of traumatic intervertebral disc extrusion in dogs. Vet Rec.

[31] Griffiths IR (1970). A syndrome produced by dorso-lateral “explosions” of the cervical intervertebral discs. Vet Rec.

[32] Kobayashi N, Asamoto S, Doi H, Ikeda Y, Matusmoto K (2003). Spontaneous regression of herniated cervical disc. Spine J..

[33] Harari J, Marks SL (1992). Surgical treatments for intervertebral disc disease. Vet Clin North Am Small Anim Pract.

[34] Brisson BA (2010). Intervertebral disc disease in dogs. Vet Clin North Am Small Anim Pract.

[35] Royal AB, Chigerwe M, Coates JR, Wiedmeyer CE, Berent LM (2009). Cytologic and histopathologic evaluation of extruded canine degenerate disks. Vet Surg.

[36] Hamilton T, Glass E, Drobatz K, Agnello KA (2014). Severity of spinal cord dysfunction and pain associated with hydrated nucleus pulposus extrusion in dogs. Vet Comp Orthop Traumatol.

